# Assessment of effectiveness measures in patients with schizophrenia initiated on risperidone long-acting therapy: the SOURCE study results

**DOI:** 10.1186/1471-244X-11-167

**Published:** 2011-10-14

**Authors:** Wayne Macfadden, Cherilyn DeSouza, Concetta Crivera, Chris M Kozma, Riad D Dirani, Lian Mao, Stephen C Rodriguez

**Affiliations:** 1 Ortho McNeil Janssen Scientific Affairs, LLC, Titusville, NJ, USA; 2 Veterans Affairs Medical Center, Kansas City, MO, USA; 3 University of South Carolina, Columbia, SC, USA; 4 Johnson & Johnson Pharmaceutical Research and Development LLC, Titusville, NJ, USA

## Abstract

**Background:**

To evaluate effectiveness outcomes in a real-world setting in patients with schizophrenia initiating risperidone long-acting therapy (RLAT).

**Methods:**

This was a 24-month, multicenter, prospective, longitudinal, observational study in patients with schizophrenia who were initiated on RLAT. Physicians could change treatment during the study as clinically warranted. Data were collected at baseline and subsequently every 3 months up to 24 months. Effectiveness outcomes included changes in illness severity as measured by Clinical Global Impression-Severity (CGI-S) scale; functional scores as measured by Personal and Social Performance (PSP) scale, Global Assessment of Functioning (GAF), and Strauss-Carpenter Levels of Functioning (LOF); and health status (Medical Outcomes Survey Short Form-36 [SF-36]). Life-table methodology was used to estimate the cumulative probability of relapse over time. Adverse events were evaluated for safety.

**Results:**

532 patients were enrolled in the study; 209 (39.3%) completed the 24-month study and 305 (57.3%) had at least 12 months of follow-up data. The mean (SD) age of patients was 42.3 (12.8) years. Most patients were male (66.4%) and either Caucasian (60.3%) or African American (23.7%). All changes in CGI-S from baseline at each subsequent 3-month follow-up visit were statistically significant (*p *< .0001), indicating improvement in disease severity. Improvements were also noted for the PSP, GAF, and total LOF, indicating improvement in daily functioning and health outcome.

**Conclusions:**

Patients with schizophrenia who were initiated on RLAT demonstrated improvements in measures of effectiveness within 3 months, which persisted over 24 months.

**Trial Registration:**

ClinicalTrials.gov: NCT00246194

## Background

Schizophrenia is a chronic debilitating mental illness with a lifetime prevalence of 1% [1], characterized by perturbations of cognition and behavior and by abnormal or limited display of emotion. Because of the severity of symptoms and the long-term, chronic pattern of schizophrenia, patients often have significant disability with serious physical, social, and economic consequences [2,3]. Major treatment goals are to maintain symptom relief, decrease relapses, increase functioning, and improve quality of life.

Long-term antipsychotic therapy is the cornerstone of schizophrenia management [4]. First-generation or conventional oral antipsychotic agents, such as fluphenazine and haloperidol, have been used for decades to treat patients with schizophrenia and are effective in reducing many symptoms of the disease. However, adverse events (AEs) associated with these drugs, including the risk of extrapyramidal symptoms (e.g., dystonias, parkinsonism, and akathisia) and tardive dyskinesia at therapeutic doses limit their use in some patients. Long-acting injectable antipsychotics (e.g., fluphenazine decanoate, haloperidol decanoate) were developed to simplify treatment and improve adherence. The pharmacokinetic profile of these agents showed reduced differences in peak and trough plasma drug levels, which allowed for more reliable drug delivery [5]. Meta-analysis of injectable versus oral therapy showed that relapse rates were significantly lower with injectable therapy [6-8].

Several of the second-generation or "atypical" agents, including risperidone, have been shown to be more effective than conventional antipsychotics [9] and have an improved safety profile with lower risk of extrapyramidal AEs and tardive dyskinesia [10-12], although the incidence of extrapyramidal AEs may vary among the atypical antipsychotics [13]. Atypical long-acting antipsychotic therapy, a relatively new treatment modality in many systems of care, is an important treatment option for many patients with chronic disease. Risperidone long-acting therapy (RLAT) is an atypical antipsychotic approved for the treatment of schizophrenia [14]. Short- and long-term studies have established the efficacy and tolerability of RLAT in patients with schizophrenia [4,15,16]. In addition, RLAT treatment is associated with low relapse and rehospitalization rates [17-22], improved treatment adherence [23], and health-related quality of life [24].

Patients with schizophrenia often have poor adherence to medication, with up to 50% of patients either partially adherent or nonadherent to medication within 1 year after discharge [25]. Nonadherence is a contributing factor for patients relapsing [26,27] and may become more important over time [7]. Use of antipsychotic medication reduces the rate of relapse in schizophrenic psychoses from 75% to 20% [28]. Strategies that improve adherence to antipsychotic therapy, such as simplification of medication regimens, monotherapy, and use of long-acting injectable therapy, may lead to improved outcomes [4,29]. Long-acting injectable therapies are convenient (i.e., patients take one fewer medication every day) and, because the injections must be administered by a health care provider, the clinical team is immediately alerted when a patient is nonadherent [20,29].

Observational studies that collect data from naturalistic clinical practice settings complement data collected from randomized controlled trials. By collecting observational data, the clinical effectiveness of RLAT on important outcomes such as health-related quality of life, disease severity, patient functionality, and tolerability can be further understood in the context of the wider spectrum of care. The Schizophrenia Outcomes-Utilization, Relapse, and Clinical Evaluation (SOURCE) study was a large-scale, prospective, observational study designed to observe effectiveness outcomes and tolerability of RLAT in real-world practice by following patients with schizophrenia initiated on RLAT for 2 years. Results of the effectiveness outcomes measures from the SOURCE study are presented herein.

## Methods

### Study design

This was a 24-month, multicenter, prospective, longitudinal, observational study (NCT00246194). A central institutional review board (IRB; Quorum Review Inc., Seattle, WA) was used to review and approve the final study protocol for all sites that participated in the study with the exception of 9 sites that required local IRB approval. This study was conducted in accordance with the ethical principles established in the Declaration of Helsinki and that are consistent with Good Clinical Practice and applicable regulatory requirements. All subjects provided written consent.

Patients eligible for enrollment were those aged 18 years and older who required treatment initiation on RLAT, had a physician-based diagnosis of schizophrenia according to the *Diagnostic and Statistical Manual of Mental Disorders*, Fourth Edition, and provided written informed consent. Patients who were at imminent risk of injury to themselves or others or of causing significant damage to property, who had a known hypersensitivity to RLAT or any of its components, or who had been treated with investigational agents within the previous 30 days were not eligible for enrollment. Women of childbearing potential who were not using an adequate method of contraception and women who were pregnant or breast-feeding were also not eligible for participation.

Patients were enrolled from 67 community mental health centers and Veterans Administration Hospitals in the United States (US) from September 2004 until January 2006, with follow-up visits through October 2007. The RLAT starting dose that was recommended to physicians was 25 mg administered every 2 weeks by deep intramuscular gluteal injection. Physicians were permitted to provide a higher RLAT dose if they deemed it necessary for their patients. After enrollment, however, specific clinical interventions were not mandated other than the initiation of RLAT at the beginning of the study; therefore, treatments for schizophrenia could be stopped, started, or changed as deemed appropriate by the patient's physician. Concomitant medications could be added to treatment regimens at the discretion of the investigator.

### Assessments

Demographic and clinical characteristics were collected at baseline and included age, gender, ethnicity, diagnosis, duration of illness, and employment status. Antipsychotic medication history during at least the past 12 months and current antipsychotic medications were recorded. Effectiveness assessments described below were performed every 3 months for 2 years. Disease severity at the time of evaluation was assessed by the Clinical Global Impression-Severity (CGI-S) [30] scale, which is a subjective measure of disease severity made by the physician on a 7-point scale ranging from 1 (normal, not at all ill) to 7 (most severely ill).

Functionality was assessed by the Personal and Social Performance (PSP) scale [31], Global Assessment of Functioning (GAF) [32], and Strauss-Carpenter Levels of Functioning (LOF) [33]. The PSP rates the patient's level of functioning during the past month in four areas: socially useful activities, personal and social relationships, self-care, and disturbing and aggressive behaviors. Physicians assign a score that ranges from 1 (lack of autonomy in basic functioning) to 100 (excellent functioning in all four areas). The GAF is a single-item rating of the patient's psychologic, social, and occupational functioning on a hypothetical continuum of mental health. Physicians rate the lowest level of function in the last week, with scores ranging from 1 (persistent danger) to 100 (superior functioning). The LOF evaluates functionality in the last month in four areas: symptoms, social contacts, work, and function. The physician rates the nine items of the LOF on a scale of 0 (worst functioning) to 4 (best functioning), with a possible maximum score of 36.

Health status was assessed with the Medical Outcomes Survey Short Form-36 (SF-36) [34], a widely used patient-reported outcomes instrument. The survey includes 36 items and evaluates health status in the past 4 weeks, in eight different areas, that can be broadly summarized as physical health (physical functioning, role-physical, body pain, general health) and mental health (vitality, social functioning, role-emotional, mental health). From these domains, physical and mental health component summary measures were also obtained. In addition, AEs and serious adverse events (SAEs) were collected during the study.

### Statistical analysis

To ensure an adequate sample size, the number of patients needed to detect meaningful changes in some of the outcome measures was estimated. A 5-point change on an individual SF-36 domain is considered clinically and socially relevant [34]. To detect a 5-point change on the most variable individual SF-36 domain score (role-physical), with 80% power and 0.05 tolerance of type 1 error, 293 evaluable patients were required, based on a two-sided paired *t *test, assuming within-subject test-retest correlation of 0.60 and standard deviation (SD) of 34 (data for the US normal subjects). This sample size also provided approximately 80% power for detection of 0.165 standardized change (effect size) in the average number of hospitalizations per year. In terms of the precision of event rate estimation (e.g., relapse rate), 293 patients would provide a rate estimate with a standard error of 3%. Assuming that approximately 50% of patients would drop out within 1 year [35,36], enrollment of 600 patients was planned.

Analysis included evaluation of baseline demographics, clinical characteristics, and functional scores. Categorical variables were summarized using frequencies and percentages. Continuous measures were summarized with mean, standard deviation, minimum, maximum, and median.

Clinical effectiveness data were analyzed in patients who had a non-missing baseline and at least one postbaseline assessment for a given effectiveness measurement and used mixed-model methodology with baseline value as a covariate, a fixed effect for time, and a random effect for center. An unstructured covariance matrix was used to model within-subject correlation. Statistical analysis software (SAS, version 9.1) was used for all analyses. All tests were two-tailed and conducted at the 5% significance level. Because of the exploratory nature of the study, no correction was made for multiplicity.

Relapse was defined as either a psychiatric hospitalization or the occurrence of a psychiatric event (defined as deliberate self-injury, suicidal or homicidal ideation that was clinically significant as determined by the investigator, or violent behavior resulting in clinically significant injury to another person or property damage). Life-table methodology was used to estimate the cumulative probability of relapse and the corresponding 95% confidence interval (CI) at each 3-month postbaseline time interval. The conditional probability of relapse for each follow-up time interval (probability of having a relapse in the current time interval for patients who were relapse free) was also calculated. Relapse time was censored at the last time interval of known status if the patient had no relapse by the end of the follow-up period, the patient withdrew from the study, or the patient was lost to follow-up.

An exploratory analysis was conducted to evaluate the impact of RLAT discontinuation among patients who had all nine visits and at least one RLAT injection record in the injection log. Effectiveness measures were compared with those receiving or not receiving RLAT. Each visit was identified as a visit during which the patient either received RLAT or did not receive RLAT. Patients were counted as having received RLAT at a visit if they had at least one record for RLAT in the injection log within 28 days prior to the visit. Because RLAT steady-state plasma concentrations are maintained at a minimum of 4 weeks after the last injection [14], the 28-day interval was chosen by the investigators to establish a time frame in which RLAT may not be able to provide adequate efficacy. Effectiveness was assessed using the CGI-S, PSP, and GAF scores collected at each visit. A mixed-model analysis of covariance was used to analyze the ranked change from baseline for each effectiveness score. The models included baseline value for the effectiveness measure, visit, an indicator variable for whether the patient was or was not receiving RLAT, and an interaction term between the visit and the RLAT indicator variable. The *a priori *hypothesis focused on the differences between patients receiving or not receiving RLAT at each visit. AEs and SAEs were summarized descriptively.

## Results

The study enrolled 532 patients; 305 (57.3%) had 12 months and 209 (39.3%) patients had 24 months of follow-up data and completed all 9 visits. Disposition of patients is summarized in Table 1.

**Table 1:** Patient disposition

	*n *(%)
**Total patients enrolled**	532
**Patients with data at 12 months**	305 (57.3)
**Patients with data at 24 months**	209 (39.3)
**Discontinued**	237 (44.5)
**Reason for discontinuation**	
Lost to follow-up	73 (13.7)
Other	65 (12.2)
Withdrawal of consent	57 (10.7)
Patient nonadherence	21 (3.9)
Adverse event	7 (1.3)
Insufficient response	6 (1.1)
Death	5 (0.9)
Patient ineligible to continue trial	2 (0.4)
Missing	1 (0.2)
**Not reported (no notification of discontinuation/no submission of 24-month data)**	86 (16.2)

The mean (SD) age was 42.3 (12.8) years, and 66.4% of patients were male. Most patients were Caucasian (60.3%) or African American (23.7%). Mean (SD) length of diagnosis was 17.9 (12.3) years. Baseline characteristics are summarized in Table 2. Of the 532 patients, 186 (36.0%) were recorded by their investigators to have received at least one antipsychotic other than RLAT after the baseline visit.

**Table 2:** Baseline demographic and illness characteristics (N = 532)

**Age, y^a^**	
Mean (SD)	42.3 (12.8)
Median (min, max)	43.2 (18, 80)
**Gender, *n *(%)**	
Male	353 (66.4)
Female	179 (33.6)
**Ethnicity, *n *(%)**	
Caucasian	321 (60.3)
African American	126 (23.7)
Hispanic	61 (11.5)
Mixed race	9 (1.7)
Other	8 (1.5)
Asian	7 (1.3)
**Duration of schizophrenia, y^b^**	
Mean (SD)	17.9 (12.3)
Median (min, max)	16.0 (0.0; 57.0)
**Schizophrenia type, *n *(%)**	
Paranoid	359 (67.5)
Undifferentiated	98 (18.4)
Disorganized	59 (11.1)
Residual	10 (1.9)
Catatonic	4 (0.8)
Missing	2 (0.4)
**Employment status, *n *(%)^c^**	
Unemployed	218 (41.0)
Disabled/long-term sick leave	134 (25.2)
Part-time	19 (3.6)
Full-time	14 (2.6)
Student	5 (0.9)
Homemaker	4 (0.8)
Retired	3 (0.6)
Missing	176 (33.1)
**Income, *n *(%)**	
<$20,000.00	239 (44.9)
$20,000 to $34,000	15 (2.8)
$35,000 to $49,999	4 (0.8)
$50,000 to $74,999	1 (0.2)
>$75,000.00	3 (0.6)
Patient refused	3 (0.6)
Patient unemployed	91 (17.1)
Missing	176 (33.1)

max, maximum; min, minimum; SD, standard deviation.

^a^*n *= 528

^b^*n *= 518

^c^Sum of percentages can be greater than 100 because patients can be in more than one group.

The most common reasons for initiating RLAT were insufficient response to previous therapy (53.8%) and lack of adherence to previous therapy (48.1%). RLAT was initiated at a dose of 25 mg in 75% of patients and at either 37.5 mg (13%) or 50 mg (11%) in the remaining patients.

### Health status

The mean (standard error [SE]) mental health component summary scores from the SF-36 was 38.0 (0.8) at baseline, 42.4 (0.9) at 12 months, and 44.5 (1.0) at the final visit. Mean scores at all postbaseline visits were statistically significantly greater than baseline (*p *< .0001). For the individual mental health domains of vitality, social functioning, role-emotional, and mental health, all postbaseline least squares (LS) means increased significantly (*p *< .005) from baseline. No statistically significant differences from baseline were observed in the physical health component summary score.

### Effectiveness outcomes

**Figure 1:**
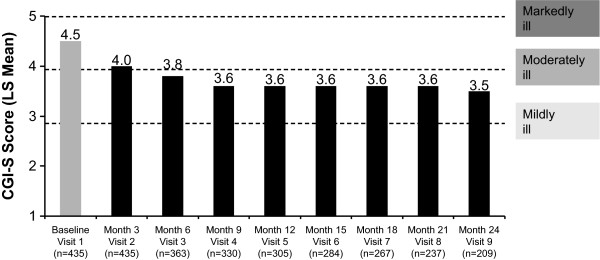
**Improvement from baseline in Clinical Global Impression-Severity (CGI-S) score**. Scores are presented as least-squares (LS) means except for the baseline value, which is unadjusted. Patients included in this analysis were those who had a baseline and at least one follow-up assessment of their CGI-S score. *Changes from each visit compared with baseline (visit 1) were significant at *p *< .0001.

The mean CGI-S scores at baseline (unadjusted) and each subsequent 3-month follow-up visit (LS means) are shown in Figure 1. All differences from baseline were statistically significant (*p *< .0001). The CGI-S score at baseline was 4.5 (marked-to-moderate illness severity) and decreased to 3.5 (moderate-to-mild illness severity) at 24 months.

**Figure 2:**
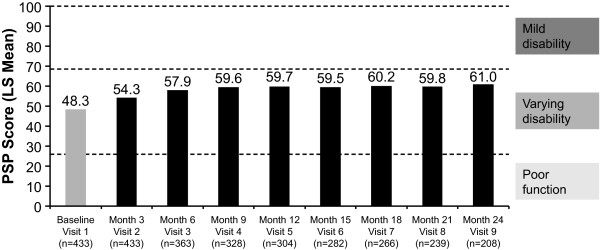
**Improvement from baseline in Personal and Social Performance (PSP) score**. Scores are presented as least-squares (LS) means except for the baseline value, which is unadjusted. Patients included in this analysis were those who had a baseline and at least one follow-up assessment of their PSP score. *Changes from each visit compared with baseline (visit 1) were significant at *p *< .0001.

The mean PSP scores at baseline (unadjusted) and each subsequent 3-month follow-up visit (LS means) are shown in Figure 2. All differences from baseline were statistically significant (*p *< .0001). The mean PSP score at baseline was 48.3 and increased to 61.0 at 24 months, indicating improvement after the initiation of RLAT.

**Figure 3:**
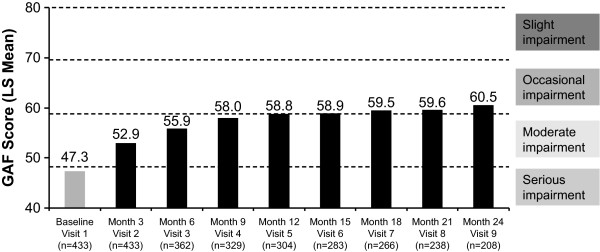
**Improvement from baseline in Global Assessment of Functioning (GAF) score**. Scores are presented as least-squares (LS) means except for the baseline value, which is unadjusted. Patients included in this analysis were those who had a baseline and at least one follow-up assessment of their GAF score. *Changes from each visit compared with baseline (visit 1) were significant at *p *< .0001.

The mean GAF scores at baseline (unadjusted) and each subsequent 3-month follow-up visit (LS means) are shown in Figure 3. All differences from baseline were statistically significant (*p *< .0001). The mean GAF score at baseline was 47.3 and increased to 60.5 at 24 months, indicating an overall significant improvement in patient functioning from serious to occasional impairment after initiation of treatment with RLAT.

**Figure 4:**
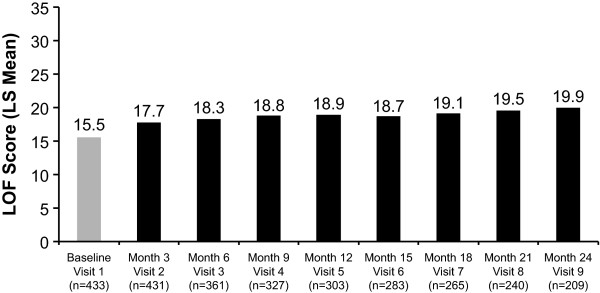
**Improvement from baseline in Strauss-Carpenter Levels of Functioning (LOF) total score**. Scores are presented as least-squares (LS) means except for the baseline value, which is unadjusted. Patients included in this analysis were those who had a baseline and at least one follow-up assessment of their LOF score. *Changes from each visit compared with baseline (visit 1) are significant at *p *< .0001.

The total LOF scores at baseline (unadjusted) and each subsequent 3-month follow-up visit (LS means) are shown in Figure 4. All differences from baseline were statistically significant (*p *< .0001). The mean total LOF scale score at baseline was 15.5 (of a maximum 36 points) and increased to 19.9 at 24 months, indicating improvement in functionality after initiation on treatment with RLAT.

### Subgroup analysis

An analysis was conducted on the effectiveness measures of CGI-S and GAF in patients who remained in the study for 24 months (n = 209 and n = 208, respectively). Mean CGI-S and GAF scores over time were evaluated, and the results on these measures were similar to what was observed for the entire sample, with significant improvement seen at the first (3-month) assessment, which persisted throughout the study.

### Relapse

**Figure 5:**
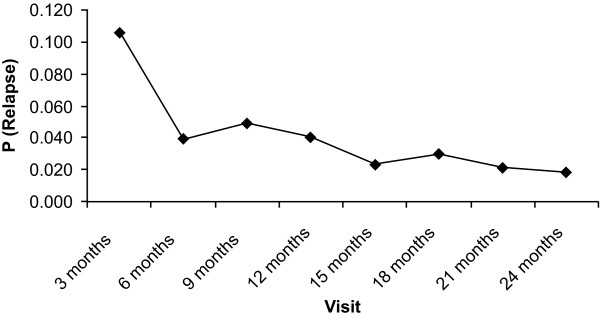
**Conditional probability of relapse by visit**. P, probability.

Half of the observed relapses occurred during the first 3 months of the study. The cumulative probability of relapse was 10.6% (95% CI: 8.2% to 13.6%) by the end of the first 3 months of the study and 28.5% (95% CI: 24.0% to 33.6%) by 24 months. Figure 5 shows the conditional probability of relapse over time by visit. The conditional probability of relapse decreased during the 24-month follow-up period (ranged from 1.8% to 4.9%).

### Exposure and discontinuation analyses

Of the 209 patients who had 24 months of data and attended all 9 visits, 202 patients had at least one documented RLAT injection recorded in the injection log and were included in the exposure and discontinuation analysis. RLAT status could not be determined for the remaining 7 patients. Table 3 presents the number and percentage of patients who received RLAT injection at each visit. At the 3-month follow-up visit, 90.1% of patients were treated with RLAT, and at the 24-month follow-up visit, 77.2% were treated with RLAT.

**Table 3:** Patients who received or did not receive RLAT who had 24 months of data and received RLAT within 28 days of the study visit

Visit	Receiving RLAT		Not Receiving RLAT	
Baseline	*n *	%	*n*	%
	202	100	0	0
3 months	182	90.1	20	9.9
6 months	182	90.1	20	9.9
9 months	173	85.6	29	14.4
12 months	167	82.7	35	17.3
15 months	164	81.2	38	18.8
18 months	163	80.7	39	19.3
21 months	160	79.2	42	20.8
24 months	156	77.2	46	22.8

**Figure 6:**
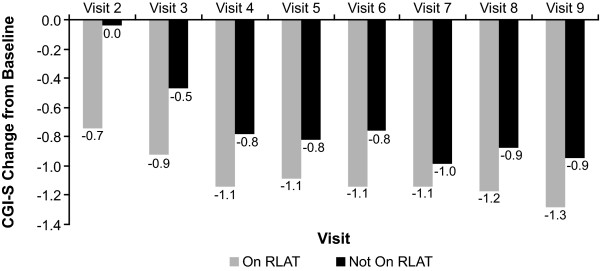
**Least-squares (LS) mean Clinical Global Impression-Severity (CGI-S) change from baseline by visit and discontinuation status**. Patients included in this analysis were those who had 24 months of data and had all nine visits and at least one RLAT injection record in the injection log. Model: CGI-S Change From Baseline = Baseline CGI-S Value + Visit + Stayed On/Dropped Off Maker + Visit*Drop Off Interaction Term. *p*-values were generated from a model using ranks of CGI-S change from baseline as the dependent variable. *All values except visit 7 were significant at *p *< .05; for visit 7, *p *= .2081.

**Figure 7:**
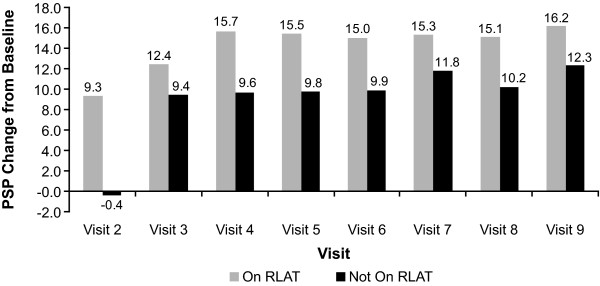
**Least-squares (LS) mean Personal and Social Performance (PSP) change from baseline by visit and discontinuation status**. Patients included in this analysis were those who had 24 months of data and had all nine visits and at least one RLAT injection record in the injection log. Model: PSP Change From Baseline = Baseline PSP Value + Visit + Stayed On/Dropped Off Maker + Visit*Drop Off Interaction Term. *p*-values were generated from a model using ranks of PSP change from baseline as the dependent variable. *All values except visit 3 were significant at *p *< .05; for visit 3, *p *= .1309.

**Figure 8:**
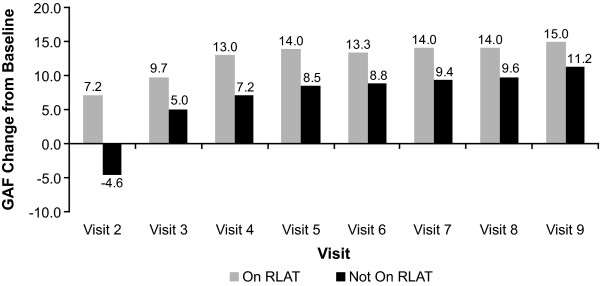
**Least-squares (LS) mean Global Assessment of Functioning (GAF) change from baseline by visit and discontinuation status**. Patients included in this analysis were those who had 24 months of data and had all nine visits and at least one RLAT injection record in the injection log. Model: GAF Change From Baseline = Baseline GAF Value + Visit + Stayed On/Dropped Off Maker + Visit*Drop Off Interaction Term. *p*-values were generated from a model using ranks of GAF change from baseline as the dependent variable. *All values were significantly different at p < .05.

Patients who continued to receive RLAT scored significantly better on measures of effectiveness (CGI-S, GAF, and PSP) than did patients who had discontinued RLAT. Patients receiving RLAT averaged approximately a 0.4-point greater decrease on the CGI-S, indicating improvements in illness severity, and approximately a 5-point greater improvement in functionality compared with patients not receiving RLAT (Figures 6, 7, 8). The tests for whether patients were receiving RLAT and the visit variable were significant at *p *< .0001 in all models. The interaction between the visit variable and the RLAT variable was not significant in any of the models.

### Adverse events

Of the 532 enrolled patients, 251 (47.2%) experienced at least one AE, and 144 (27.1%) experienced at least one SAE over the 24-month follow-up period. AEs reported in more than ≥5% of patients were psychotic disorder (9.4%), anxiety (7.7%), depression (7.5%), suicide ideation (6.0%), insomnia (5.6%), and schizophrenia (4.5%). Five patients died during the study; three due to cardiac failure, one due to chronic obstructive pulmonary disease and cardiac failure, and one due to complications from the flu. The deaths were not related to study medication.

## Discussion

The results of this observational study found that patients with schizophrenia had reduced illness severity (as measured by CGI-S) and improved clinician-rated functioning (as measured by GAF, PSP, and LOF) after 3 months of treatment. These changes were maintained for 24 months. Atypical antipsychotic agents have been shown to have a positive impact on factors most associated with quality of life (e.g., negative and affective symptoms and drug tolerability); thus treatment with an atypical agent such as RLAT may lead to improved health status.

Because this was an open-label observational study without a comparator group, its limitations require that these data be interpreted cautiously and not be overly generalized. Initiation of RLAT at the beginning of the study was the only clinical mandate, and treatments for schizophrenia including additional concomitant medications could be stopped, started, or changed at the investigator's discretion. Therefore, patients could have been using additional antipsychotics during the study, and the effectiveness observed may have been derived from different medications other than RLAT. In the total population, 36% of patients were recorded by their investigators as receiving additional antipsychotics other than RLAT. However, since the accuracy of information depended on clinician reports, patients other than those documented may have been using additional antipsychotics.

RLAT and other long-acting injectables are thought to improve treatment adherence [23], hence, the high dropout rate was of concern as it may have affected the results. However, because this was an observational study, patients were not obligated to participate in all 9 visits. Although the sample size decreased by 60% over the course of the current study, high dropout rates are common in observational studies with extended follow-ups.

To address these potential biases, an exploratory subgroup analysis was conducted in patients who had 24 months of data and received RLAT within 28 days of the study visit in order to evaluate a patient population in which RLAT use was known. In this population, patients receiving RLAT had greater improvements in effectiveness measures than patients not receiving RLAT. The results within this cohort were almost identical to those observed for the entire sample, which increases the validity of these data.

Although the data from this study should be interpreted cautiously, the results compare favorably with previously reported studies evaluating RLAT in patients with schizophrenia. Similar to previously reported data [25,37], a significant decrease of 1.3 points in mean CGI-S score from baseline (4.5 points indicative of marked-to-moderate illness severity) to 24 months (3.2 points indicative of moderate-to-mild illness severity) was noted. Significant improvements of up to 22% were also observed in global functioning, corresponding to a change from serious to moderate disability. Furthermore, in this study, the 2-year estimated cumulative relapse rate was 28.5%, which compares favorably with literature-reported relapse rates. A 1-year relapse rate of 18% and 2-year relapse rate of 23% were observed in a study of first-episode schizophrenic patients receiving RLAT compared with 50% and 75%, respectively, for patients receiving oral risperidone [38]. In a post hoc comparison of responder patients in South Africa, relapse rates at 24 months were 9.3% for patients receiving RLAT and 42.1% for those receiving oral risperidone or haloperidol [33]. Stable schizophrenic patients who were randomized to two fixed doses of RLAT had 1-year incidence of relapse of 15% and 22% [17].

Improvement in health status, as assessed by the SF-36 mental health domains and summary measure, was also observed at 3 months and maintained for 24 months. Significant and sustained improvements in negative symptoms and positive changes in mental health quality of life have been reported at 1 month and up to 6 months after open-label treatment with RLAT [39-42]. Improved health status with RLAT was also observed by Nasrallah et al. in a double-blind study comparing patients receiving RLAT with those receiving a placebo [24].

The SOURCE study, to the best of our knowledge, is the first 24-month observational study in the US to follow these effectiveness measures in schizophrenic patients initiated on RLAT. Additionally, the electronic Schizophrenia Treatment Adherence Registry (eSTAR), an ongoing multinational, observational registry study, has been evaluating outcomes in patients with schizophrenia. After 24 months of follow-up in eSTAR in Spain, RLAT use was associated with increased efficacy [23]. Additional eSTAR results on pooled data from six and eight other countries participating in eSTAR showed significant improvement in CGI-S and GAF scores [43,44].

## Conclusions

Although efficacy and safety data from randomized controlled clinical trials are the mainstay of regulatory decision-making for drug marketing approval, interest in well-designed postapproval observational and registry studies has increased and the results from these studies may have broader applicability [45]. Clinical management of patients with schizophrenia is lifelong and requires family, social, and therapeutic interventions, including antipsychotic therapy, to stabilize and support patients. In the SOURCE study, initiation and continuation of RLAT may support the long-term effectiveness goals by reducing disease severity and improving function and mental health-related quality of life.

## Competing interests

WM is a former employee of Ortho-McNeil Janssen Scientific Affairs, LLC. CD declares that she has no competing interests. CC, RD, and SCR are employees of Ortho-McNeil Janssen Scientific Affairs and Johnson & Johnson stockholders. CMK was contracted by Ortho-McNeil Janssen Scientific Affairs, LLC, to perform the statistical analysis for this manuscript. LM is an employee of Johnson & Johnson Pharmaceutical Research and Development and a Johnson & Johnson stockholder.

## Authors' contributions

CD, CC, CMK, RDD, LM, and SCR contributed to the conception and design, acquisition of data, analysis and interpretation of data, and drafting of the manuscript and its critical revision for important intellectual content.

WM was involved in the interpretation of data and in the critical drafting and revising of the manuscript for important intellectual content.

All authors read and approved the final manuscript.

## Pre-publication history

The pre-publication history for this paper can be accessed here:

http://www.biomedcentral.com/1471-244X/11/167/prepub
